# Spatiotemporal Clustering of *Mycobacterium tuberculosis* Complex Genotypes in Florida: Genetic Diversity Segregated by Country of Birth

**DOI:** 10.1371/journal.pone.0153575

**Published:** 2016-04-19

**Authors:** Marie Nancy Séraphin, Michael Lauzardo, Richard T. Doggett, Jose Zabala, J. Glenn Morris, Jason K. Blackburn

**Affiliations:** 1 Department of Medicine, Division of Infectious Diseases and Global Medicine, College of Medicine, University of Florida, Gainesville, FL, United States of America; 2 Emerging Pathogens Institute, University of Florida, Gainesville, FL, United States of America; 3 Department of Epidemiology, College of Public Health and Health Professions, College of Medicine, University of Florida, Gainesville, FL, United States of America; 4 Bureau of Communicable Diseases, Tuberculosis Control Section, Florida Department of Health, Tallahassee, FL, United States of America; 5 Department of Geography, Spatial Epidemiology & Ecology Research Laboratory, University of Florida, Gainesville, FL, United States of America; Johns Hopkins Bloomberg School of Public Health, UNITED STATES

## Abstract

**Background:**

Tuberculosis (TB) is caused by members of the *Mycobacterium tuberculosis* complex (MTBC). Although the MTBC is highly clonal, between-strain genetic diversity has been observed. In low TB incidence settings, immigration may facilitate the importation of MTBC strains with a potential to complicate TB control efforts.

**Methods:**

We investigated the genetic diversity and spatiotemporal clustering of 2,510 MTBC strains isolated in Florida, United States, between 2009 and 2013 and genotyped using spoligotyping and 24-locus MIRU-VNTR. We mapped the genetic diversity to the centroid of patient residential zip codes using a geographic information system (GIS). We assessed transmission dynamics and the influence of immigration on genotype clustering using space-time permutation models adjusted for foreign-born population density and county-level HIV risk and multinomial models stratified by country of birth and timing of immigration in SaTScan.

**Principal Findings:**

Among the 2,510 strains, 1,245 were reported among foreign-born persons; including 408 recent immigrants (<5 years). Strain allelic diversity (*h*) ranged from low to medium in most locations and was most diverse in urban centers where foreign-born population density was also high. Overall, 21.5% of cases among U.S.-born persons and 4.6% among foreign-born persons clustered genotypically and spatiotemporally and involved strains of the Haarlem family. One Haarlem space-time cluster identified in the mostly rural northern region of Florida included US/Canada-born individuals incarcerated at the time of diagnosis; two clusters in the mostly urban southern region of Florida were composed predominantly of foreign-born persons. Both groups had HIV prevalence above twenty percent.

**Conclusions/Significance:**

Almost five percent of TB cases reported in Florida during 2009–2013 were potentially due to recent transmission. Improvements to TB screening practices among the prison population and recent immigrants are likely to impact TB control. Due to the monomorphic nature of available markers, whole genome sequencing is needed to conclusively delineate recent transmission events between U.S. and foreign-born persons.

## Introduction

Tuberculosis (TB) continues to present a major global public health challenge. In 2014, 9.6 million people developed the disease and 1.5 million died [1]. An estimated two billion of the world population is latently infected, and over their lifetime, five to ten percent will progress to active disease [2]. The bulk of TB morbidity and mortality is concentrated in low and middle income countries [1]. Nevertheless, aided by population movement and migration, TB also presents a challenge in high income countries [3–6]. The United States (U.S.) is a low-incidence country where immigration has had a substantial impact on TB epidemiology [3,7,8]. Public health efforts such as contact tracing and prophylactic treatment of latent infection have led to a substantial decrease in TB incidence over the past 20 years; from 10.4 per 100,000 in 1992 to 3.0 per 100,000 in 2014 [7]. This steady decline, however, obscures the substantial burden among foreign-born persons whom continue to account for a larger proportion of the incident cases in the U.S. [7]. As incidence is decreasing overall in the general U.S. population, TB is increasingly concentrated within high-risk U.S.-born individuals [9–11] and immigrant subgroups in large urban centers [12]. In such settings prompt identifications of local transmission is important to the continued efforts towards TB elimination.

Spacer oligonucleotide typing (Spoligotyping) and mycobacterial interspersed repetitive units variable number tandem repeats (MIRU-VNTR) used together have been instrumental to the detection of Mycobacterium *tuberculosis* complex (MTBC) outbreaks in the community by classifying strains into clusters of isolates with identical genotype patterns, with clustering serving as a proxy measure for transmission [13,14]. In the U.S., universal genotyping of culture-confirmed TB cases by both spoligotyping and MIRU-VNTR has been available since 2004 [15]. Evidence from a number of studies, however, suggest that within immigrant groups from high TB burden countries and local-born populations from enclosed rural regions, MTBC genotype clusters may result from both founder’s effect and local transmission [14,16,17]. A number of studies have used a combination of genotyping and spatial scan statistics to quantify MTBC transmission in heterogeneous settings [18–20]. Indeed, tracking genotype clusters in space and time may provide a better picture of MTBC transmission dynamics.

In this study we used space-time scan statistics in SaTScan [21], to identify foci of M. *tuberculosis* genotype clusters due to recent transmission in Florida, U.S. and assess the influence of foreign-birth on clustering. Spatiotemporal clusters were evaluated in relation to the spatial distribution of M. *tuberculosis* genetic diversity in the State. In 2014, almost 20.0% of the more than 19.8 million residents in Florida were foreign-born [22]. During the same year, TB incidence was 3.0 per 100,000 and over half of the cases occurred in the foreign-born population [7]. The foreign-born population in Florida is localized in the Southern regions of the State, including the largely urban counties of Miami-Dade, Broward and Palm Beach [23]. However, the spatial distribution and genetic diversity of M. *tuberculosis* in Florida is unknown. As TB incidence is decreasing in the US, so too is funding allocated to TB control. State TB control programs have to focus limited resources to targeting high-risk groups and areas of active TB transmission [24,25]. Studies combining geospatial scan statistics with molecular markers of TB transmission and epidemiological data will help public health officials focus limited resources to areas where they are most needed.

## Materials and Methods

### Study Population

As part of the MTBC genotyping surveillance program, at least one isolate from every culture-confirmed TB case in the US is genotyped by spoligotype and 24-locus MIRU using standardized methods [15]. The genotyping data is linked to the National Tuberculosis Information Management System (TIMS) using a unique patient identifier so that initial drug susceptibility profile, clinical, socio-demographic and risk factor data are available for each genotyped isolate [15]. From January 1, 2009 to December 31, 2013, spoligotype patterns in octal designation, 24-loci MIRU-VNTR, patient residential zip code and age at time of diagnosis, pre-treatment drug resistance profile, HIV status, history of incarceration, and country of birth were available for 2,531 culture confirmed TB cases.

### Strain Classification and Spatial Mapping

The 2,531 isolates were classified into 74 strain families and sublineages using the web application ***MIRU-VNTR****plus* on the spoligotype and 24-locus MIRU-VNTR data [26]. Loci were ordered as reported in Mazars et al., 2001 [27] and CDC notations used for ambiguous and indeterminate sites. Strain matching was allowed within up to four locus difference, using the categorical genetic distance measure. We downloaded a polygon of the five-digit Florida ZIP Code Areas from the Florida Geographic Data Library (FGDL), current as of 2012 [28]. Using the geographical coordinates at the centroids of each polygon, we created a GIS database by geocoding each reported and genotyped TB case to the patient’s five-digit residential zip code at the time of diagnosis. We assumed no substantial change in zip code shapefile over the study period.

### Spatial Descriptive Statistics, Genotyping Coverage and Genotype Diversity

We evaluated bias in genotyping compared to culture-confirmed cases by computing the yearly unweighted spatial mean centers (the average X and Y coordinates) for the reported and genotyped data and examined yearly directional trends within one standard deviation of the means by computing the standard deviation ellipses and standard distances for zip code centroids using the directional distribution tool in ArcGIS Spatial Statistics Tools (ESRI) [29]. To compare the mean centers and directional distribution measures for the two GIS databases, we overlaid them on a map of Florida. A shift in the yearly spatial mean centers would represent a bias in reported culture-confirmed TB cases compared to cases that were eventually genotyped. We calculated the genotyping coverage as the proportion of the culture-confirmed TB cases that were genotyped for each zip code location using Geospatial Modelling Environment v7.2.1 [30].

We measured the overall 24-Locus MIRU-VNTR discriminatory power for the sample using the Hunter-Gaston discriminatory index (HGDI)[31], calculated using the equation:

$$HGDI=1-\left[\frac{1}{N\left(N-1\right)}\;\sum_{j=1}^{s}n_{j}\;\left(n_{j}-1\right)\right]$$

where *N* is the total number of strains in the sample, *s* is the total number of different MIRU-VNTR patterns, and *n*_j_ is the number of strains sharing the same *j*th pattern. We measured the MIRU-VNTR allelic diversity (*h*) at each of the 24 different loci according to the equation:

$$h=1-\sum x_{i}^{2}$$

where *x*_i_ is the frequency of the *ith* allele at the locus [32]. In computing the allelic diversity, we removed indeterminate and ambiguous sites; thus the total number of isolates analyzed was 2,414. To assess the spatial distribution of MTBC genetic diversity, we computed an average allelic diversity at the zip code centroid using the same formula with *x*_*i*_ representing the frequency of the *ith* allele at that location.

### MTBC Spatiotemporal Clusters and TB Transmission

We grouped the strains into major lineages and sublineages: Beijing, East-African-Indian (EAI), Haarlem, Latin-American-Mediterranean (LAM), T, X, S, U, and Central-Asian (CAS) and M. *Bovis*. Strains of low frequencies (Africanum, Manu1, Manu2, H37Rv, and Zero) were grouped into the “other” category, while undefined strains were analyzed separately. Within each sublineage category, we defined state-based genotype clusters as two or more cases with identical spoligotype and 24-locus MIRU-VNTR profile. We used the discrete retrospective space-time permutation model implemented in SaTScan v9.4 to test for high rates of genotype clusters that also cluster in space and time [21]. The Bernoulli space-time model would have been most appropriate; however, it does not allow for covariate adjustment [21]. As we lacked information on the at-risk population at a resolution comparable to the genotyped case data, we were unable to implement the Poisson space-time statistics as previously used to identify clusters of recent TB transmission in the U.S. [18]. The space-time permutation model is ideal when the information on the population at risk is not available and is described in detail elsewhere [33]. Briefly, the space-time permutation model derives case expectations under the assumption of independent spatial interaction between case dates and locations. To operationalize the space-time permutation model, the study area is scanned using cylinders of varying sizes, with the base representing space and the height representing time. The number of cases inside the cylinders is compared to the case expectations outside by Poisson generalized linear ratio (GLR), where the cylinder that maximizes the GLR is considered the most likely cluster and additional significant clusters are defined as secondary [33]. We identified space-time clusters at a maximum temporal and spatial window of 20.0% and 50.0% of the study period and case data, respectively after testing different temporal and spatial window combinations with no significant changes in cluster size, location or test statistics. We made no distinction between primary or secondary clusters. We only allowed one set of geographical coordinates per location and no overlapping clusters. Statistically significant clusters were evaluated at a p-value ≤0.05, using 999 random Monte Carlo permutations under the null assumption of complete spatial randomness.

To account for differences in the spatial distribution of foreign-born individuals and HIV rates in Florida, we first ran unadjusted space-time permutation models and then models adjusted for the binary indicators of county-level risk of HIV and foreign-born population density [34]. Covariates were considered to affect the unadjusted clusters if they reduced the test statistic, which would suggest that part of the excess cases within the unadjusted cluster could be explained by the covariate [34]. Single year HIV rates and estimates of the proportion of individuals 5-years and older who speak a language other than English at home, as a proxy measure of foreign birth covering the period 2009 to 2013 were obtained from *FloridaCharts* [23]. We computed the rate change for each county and the state, comparing 2009 rates to rates reported for 2013. We created binary indicators of county-level HIV risk and foreign-born population density respectively by comparing a county’s rate change between the two time periods to that of the state. A county was coded as having an elevated HIV risk and/or a high density of foreign-born individuals, if the rate change for these two indicators increased in comparison to the state’s between 2009 and 2013.

### Multinomial Spatiotemporal Clusters

In cases where we detected statistically significant space-time permutation clusters, we ran multinomial space-time models to assess the influence of country of birth on MTBC genotype clustering using the same settings as for the space-time permutation models [21]. Self-reported country of birth and number of years since immigration were available for all foreign-born cases. Based on the frequency of observations, we created a five-level indicator which grouped reported country of birth into US/Canada, Haiti, Latin America, South America and Other. Timing of immigration was coded as <5 years and ≥ 5 years. The multinomial scan statistics identify zip codes with an increased occurrence of MTBC genotype clusters from particular populations based on birth country [35].

### Ethical Clearance

The data were collected as part of public health practice and thus participants were not consented. The use of the data for this study was approved by the Institutional Review Boards (IRB) of the University of Florida and the Florida Department of Health, respectively, and all data were de-identified before analyses.

## Results

Between 2009 and 2013, a total of 3,739 TB cases were reported to the Florida Department of Health (FDOH). We excluded 879 observations; 808 culture negative cases and 71 cases without zip code information. Of the remaining culture-confirmed TB cases, 2, 531 (88.5%) were genotyped. The period January 1, 2009 to December 31, 2013 represented the time period during which genotyping coverage was ideal in Florida due to improvement to the surveillance system. We observed the lowest number of genotyped cases in 2009 and 2013, while a similar number of reported cases were genotyped during 2010, 2011 and 2012 (Table 1). Overall, 46.3% of genotyped cases clustered i.e. two or more cases shared identical spoligotyping and 24-locus MIRU-VNTR patterns. Across the five-year period, genotyped cases differed significantly by the number of clustered cases, history of incarceration and country of birth. Among those born outside the U.S., genotyping did not differ significantly by timing since immigration to the U.S. In addition, there was no significant difference by gender, age at diagnosis, HIV status or history of homelessness.

**Table 1:** Characteristics of Genotyped Tuberculosis Cases in Florida, 2009–2013.

	Year of Isolation						
Characteristics	2009 (n = 382)	2010 (n = 573)	2011 (n = 560)	2012 (n = 518)	2013 (n = 498)	Total (n = 2531)	P-value^¥^
Clustered Cases							
No	183 (47.9)	333 (58.1)	291 (52.0)	279 (53.9)	274 (55.0)	1360 (53.7)	0.0304
Yes	199 (52.1)	240 (41.9)	269 (48.0)	239 (46.1)	224 (45.0)	1171 (46.3)	
Gender							
Male	257 (67.3)	376 (65.6)	368 (65.7)	331 (63.9)	308 (61.9)	1640 (64.8)	0.4795
Female	125 (32.7)	197 (34.4)	192 (34.3)	187 (36.1)	190 (38.2)	891 (35.2)	
Age							
≤24 Years	42 (11.0)	67 (11.7)	60 (10.7)	52 (10.0)	53 (10.6)	274 (10.8)	0.7173
25–44 Years	138 (36.1)	188 (32.8)	162 (28.9)	155 (30.0)	153 (30.7)	796 (31.5)	
45–64 Years	134 (35.1)	210 (36.7)	226 (40.4)	203 (39.2)	195 (39.2)	968 (38.3)	
≥65 Years	68 (17.8)	108 (18.9)	112 (20.0)	108 (20.9)	97 (19.5)	493 (19.5)	
Origin							
US/Canada	199 (52.1)	286 (45.7)	256 (45.7)	272 (52.5)	216 (43.4)	1229 (48.6)	0.0018
Haiti	54 (14.1)	71 (12.4)	78 (13.9)	55 (10.6)	65 (13.1)	323 (12.8)	
Latin America	52 (13.6)	75 (13.1)	53 (9.5)	54 (10.4)	56 (11.2)	290 (11.5)	
South America	47 (12.3)	65 (11.3)	78 (13.9)	67 (12.9)	69 (13.9)	326 (12.9)	
Other	30 (7.9)	76 (13.3)	95 (17.0)	70 (13.5)	92 (18.5)	363 (14.3)	
Time in the US*							
< 5 Years	66 (37.7)	95 (34.4)	86 (29.7)	74 (31.1)	91 (32.9)	412 (32.7)	0.4250
≥ 5 Years	109 (62.7)	181 (65.6)	204 (70.3)	164 (68.9)	186 (67.2)	844 (67.0)	
HIV Status							
Negative	249 (65.2)	410 (71.6)	393 (70.2)	371 (71.6)	382 (76.7)	1805 (71.3)	0.1123^€^
Positive	63 (16.5)	76 (13.3)	76 (13.3)	56 (10.8)	65 (13.1)	343 (13.6)	
Refused	35 (9.2)	43 (7.5)	43 (7.5)	53 (10.2)	37 (7.4)	211 (8.3)	
Missing	35 (9.2)	44 (7.7)	44 (7.7)	38 (7.3)	14 (2.8)	172 (6.8)	
History of Incarceration							
No	355 (92.9)	552 (96.3)	545 (97.3)	496 (95.8)	477 (95.8)	2425 (95.8)	0.0217
Yes	27 (7.1)	21 (3.7)	15 (2.7)	22 (4.3)	21 (4.2)	106 (4.2)	
History of Homelessness							
No	353 (92.4)	514 (89.7)	502 (89.6)	455 (87.8)	447 (89.8)	2271 (89.7)	0.1473
Yes	29 (7.6)	49 (8.6)	53 (9.5)	57 (11.0)	48 (9.6)	236 (9.3)	
Missing	0 (0.0)	10 (1.8)	5 (0.9)	6 (1.2)	3 (0.6)	24 (1.0)	

Notes:

*Among Foreign-born cases. We lacked data on timing of immigration for 3 cases;

^¥^ P-value for chi-square test of equal proportion across the five year study period;

^€^ among cases with known HIV status and those who refused the test.

Descriptive Analyses were conducted using SAS v9.4 (Cary, NC, USA).

### Genotyping Coverage and MTBC Genetic Diversity

**Fig 1:**
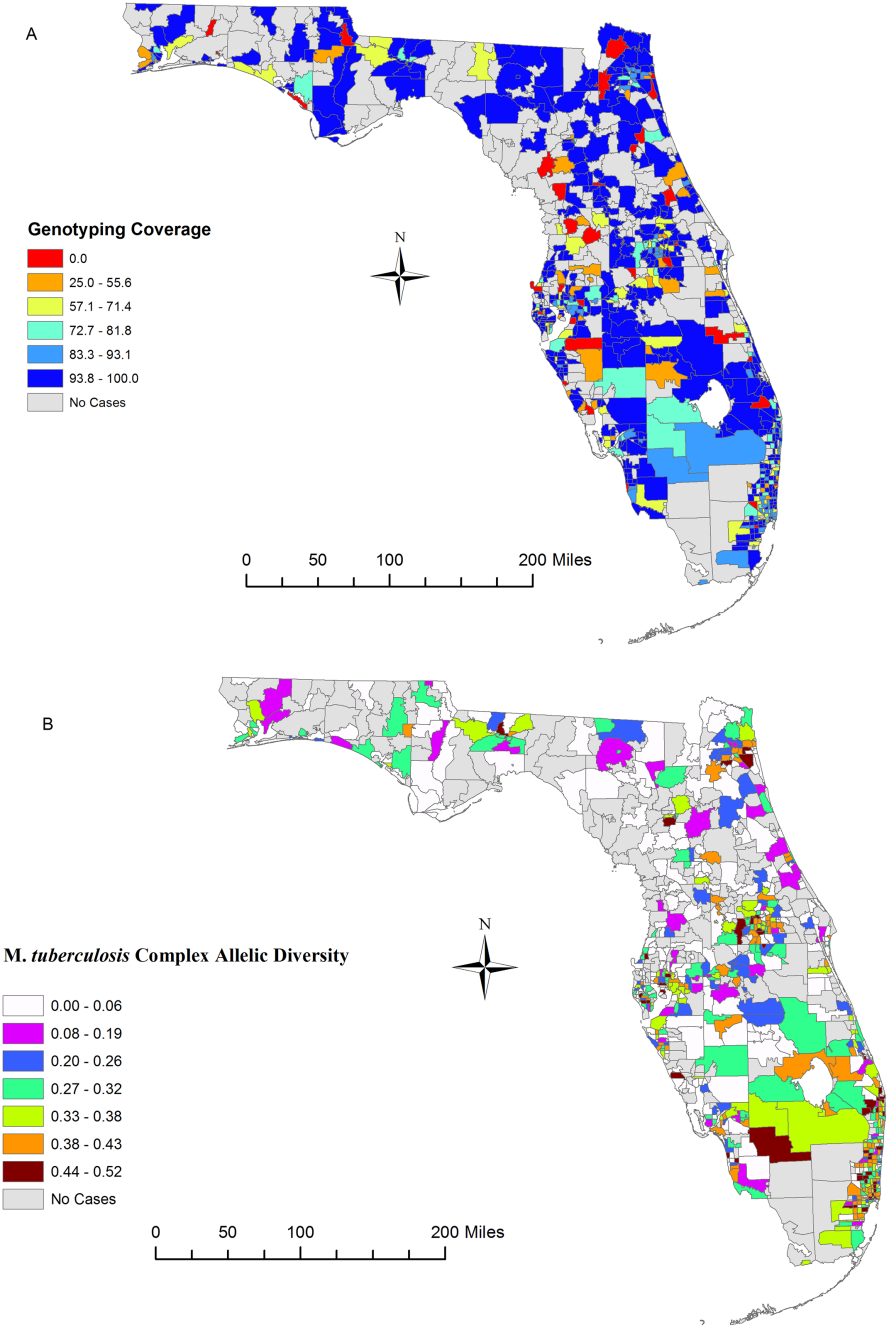
Spatial Distribution of *Mycobacterium tuberculosis complex* Allelic Diversity in Florida. Maps show genotyping coverage as a percent of reported cases that were genotyped (Panel A) and the allelic diversity of the different sublineages (Panel B) isolated in different study locations from 2009 to 2013. Allelic diversity shown is an average for the 24-Locus MIRU-VNTR loci isolated at a local. The smaller the number the less diverse the M. *tuberculosis* complex population diversity is at that location. Base map layer reprinted from [28] under a CC BY license, with permission from University of Florida GeoPlan Center, original copyright 2012.

The 2,531 isolates were categorized into 1,644 distinct 24-locus MIRU-VNTR patterns consisting of 291 genotype clusters of two or more isolates. The discriminatory index for the sample was very high (HGDI = 0.997). The final sample size for the genetic diversity analyses consisted of 2,510 observations; 21 genotyped observations were excluded as they could not be geocoded. Genotyping coverage over the five year study period was low in some locations where we observed a percentage of culture-confirmed cases genotyped ranging from 0.0% to 25.0% (Fig 1A). Overall, genotyping coverage reached above 80.0% for most parts of the study region. In addition, the spatial distribution of genotyping efforts, based on spatial descriptors, showed little difference from the distribution of reported TB cases in the study region over the five-year study period when comparing the reported culture-confirmed cases to the genotyped cases (S1 Fig). In Table 2 we show the allelic diversity (*h*) for each of 24 MIRU-VNTR loci. The diversity ranged from 0.09 for the MIRU 27 locus to 0.824 for the MIRU 4156 locus. Four loci (MIRU 02, MIRU 20, MIRU 24, MIRU 27) exhibited low level of diversity (*h* <0.30). We have provided a supplemental table showing the sublineage specific allelic diversity for isolates included in this study (S1 Table). Some of the sublineages were highly diverse at the loci, while most of the others were completely monomorphic (0.00 > *h* <0.30). In Fig 1B we show the spatial distribution of average allelic diversity (*h*) of the MTBC strain families isolated during the study period. Although a number of locations did not report cases during the study period, allelic diversity ranged from low to moderate (0.00 > *h* < 0.30) in most locations. Diversity was especially high (*h* > 0.40) in the Southeast, East Central and Northeast Regions, with the highest level of genetic diversity observed in known foreign-born population hubs in Central and South Florida.

**Table 2:** Allelic Diversity of Clinical M. tuberculosis Complex Isolates in Florida, 2009–2013.

MIRU-VNTR Locus	Number of Isolates with Indicated MIRU Allele											Allelic Diversity Index
	0	1	2	3	4	5	6	7	8	9	10	
MIRU 02		218	2146	49	1							0.20
MIRU 04	72	24	2026	101	27	122	23	9	4	6		0.29
MIRU 10	5	9	201	714	967	449	44	6	19			0.71
MIRU 16	5	116	412	1713	165	3						0.46
MIRU20	19	242	2148	5								0.20
MIRU 23	9	22	8	201	45	1438	664	19	8			0.56
MIRU24	3	2194	215	2								0.17
MIRU 26	2	13	215	160	374	1276	179	170	22	3		0.67
MIRU 27	6	22	51	2298	33	3	1					0.09
MIRU 31	1		318	1608	218	238	30	1				0.52
MIRU 39	6	44	1985	320	46	1	12					0.31
MIRU 40	16	333	387	1179	329	99	44	19	6	2		0.70
MIRU 424	25	213	1232	309	572	57	6					0.66
MIRU 577	5	1	150	804	1373	70	9	2				0.56
MIRU 1955	94	128	574	896	497	140	46	10	15	14		0.76
MIRU 2163b		28	626	450	484	431	243	57	46	47	2	0.82
MIRU 2165	1	33	681	1208	394	22	34	29	1	7	4	0.64
MIRU 2347	3	3	229	212	1944	22	1					0.34
MIRU 2401	3	391	874	8	1120	7	11					0.63
MIRU 2461	4	214	1951	49	62	37	88	9				0.34
MIRU 3171	4	132	135	2034	41	67	1					0.28
MIRU 3690	25	173	563	1313	210	77	17	26	6	4		0.64
MIRU 4052	57	246	1119	900	87	3	2					0.63
MIRU 4156	116	6	26	103	247	389	410	725	292	100		0.82

**Notes**: Indeterminate (“_”) and ambiguous sites (“%“) were removed from the calculations for genetic diversity; the total sample used is n = 2,414.

### Space-time Genotyping Clustering

**Fig 2:**
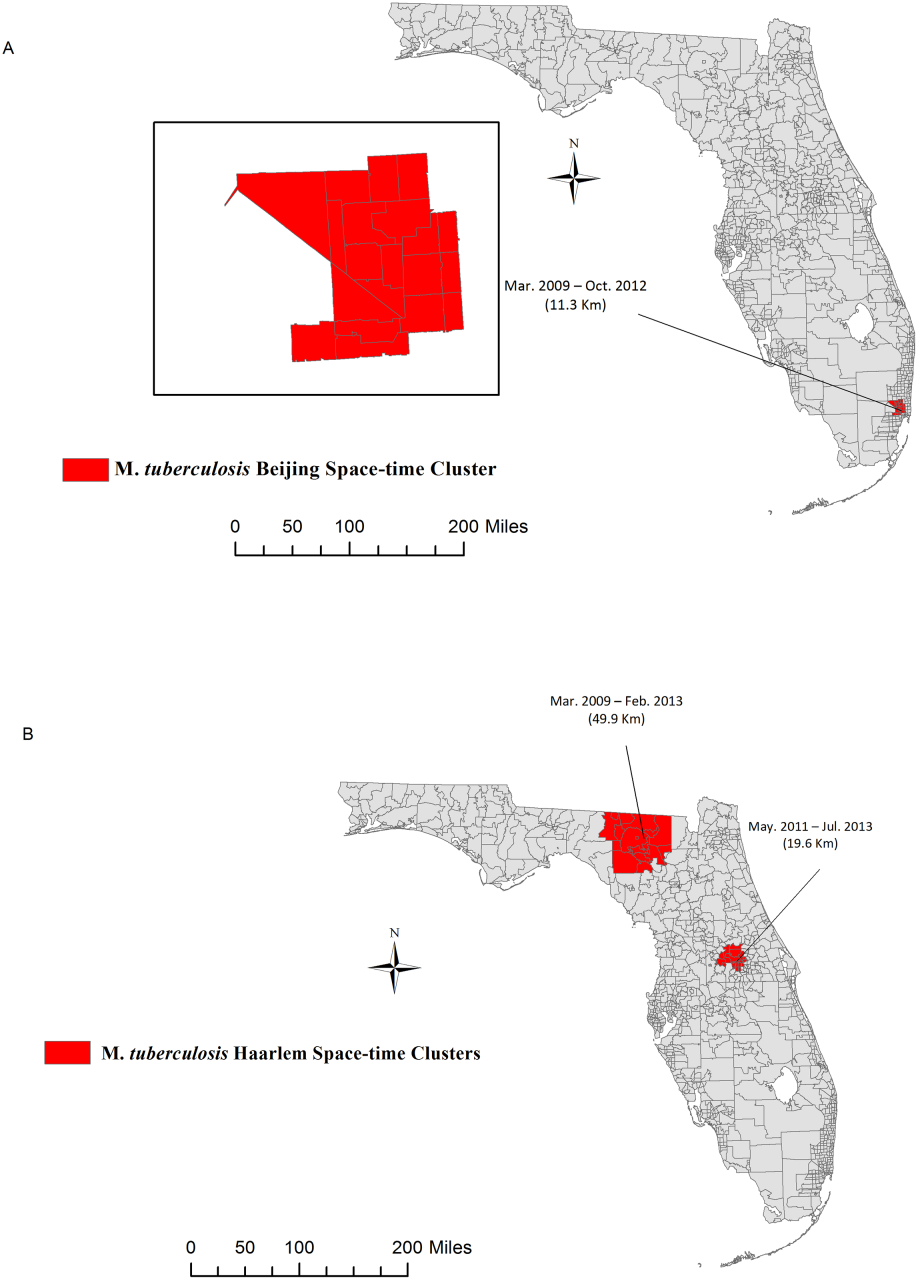
Spatiotemporal Clustering of Mycobacterium *tuberculosis* complex lineages. Maps show the locations of significant space-time clusters of M. *tuberculosis* Beijing (Panel A), and Haarlem (Panel B) sublineages in Florida, 2009–2013. Clusters were adjusted for county-level foreign-born population density and HIV risk. Adjusted Beijing Clusters were non-significant and are not shown). Base map layer reprinted from [28] under a CC BY license, with permission from University of Florida GeoPlan Center, original copyright 2012.

We ran twelve space-time permutation models including only genotype clustered strains: 107 Beijing strains, 17 M. *Bovis* strains, 6 CAS strains, 32 EAI strains, 250 Haarlem strains, 179 LAM strains, 31 S strains, 178 T strains, 32 U strains, 282 X strains, 4 others and 43 undefined, respectively. We identified one significant space-time cluster of the Beijing family in the Southeast Region of the state which persisted from March 2009 to October 2012 (Fig 2A). However, the cluster was no longer significant once we adjusted for county-level HIV risk and foreign-born population density. We detected two significant clusters of the Haarlem sublineage in North Central and East Central Florida (Fig 2B**)**. These clusters persisted even after adjusting for both county-level HIV risk and foreign-born population density. In addition, cluster sizes, location or test statistics did not vary with varying combination of spatial or temporal windows. Of the 250 cases involved in the Haarlem genotype cluster, 163 were U.S.-born, 29 were recent immigrants (<5 years) and 58 had lived in the U.S. at least five years. Based on the adjusted space-time permutation models, potential recent TB transmission in Florida involved Haarlem strain families and was estimated at 15.60% overall; 21.47% (35/250) among U.S.-born cases, 5.17% (3/58) among foreign-born persons in the U.S. ≥5 years and 3.45% (1/29) among recent arrivals (<5 years).

**Fig 3:**
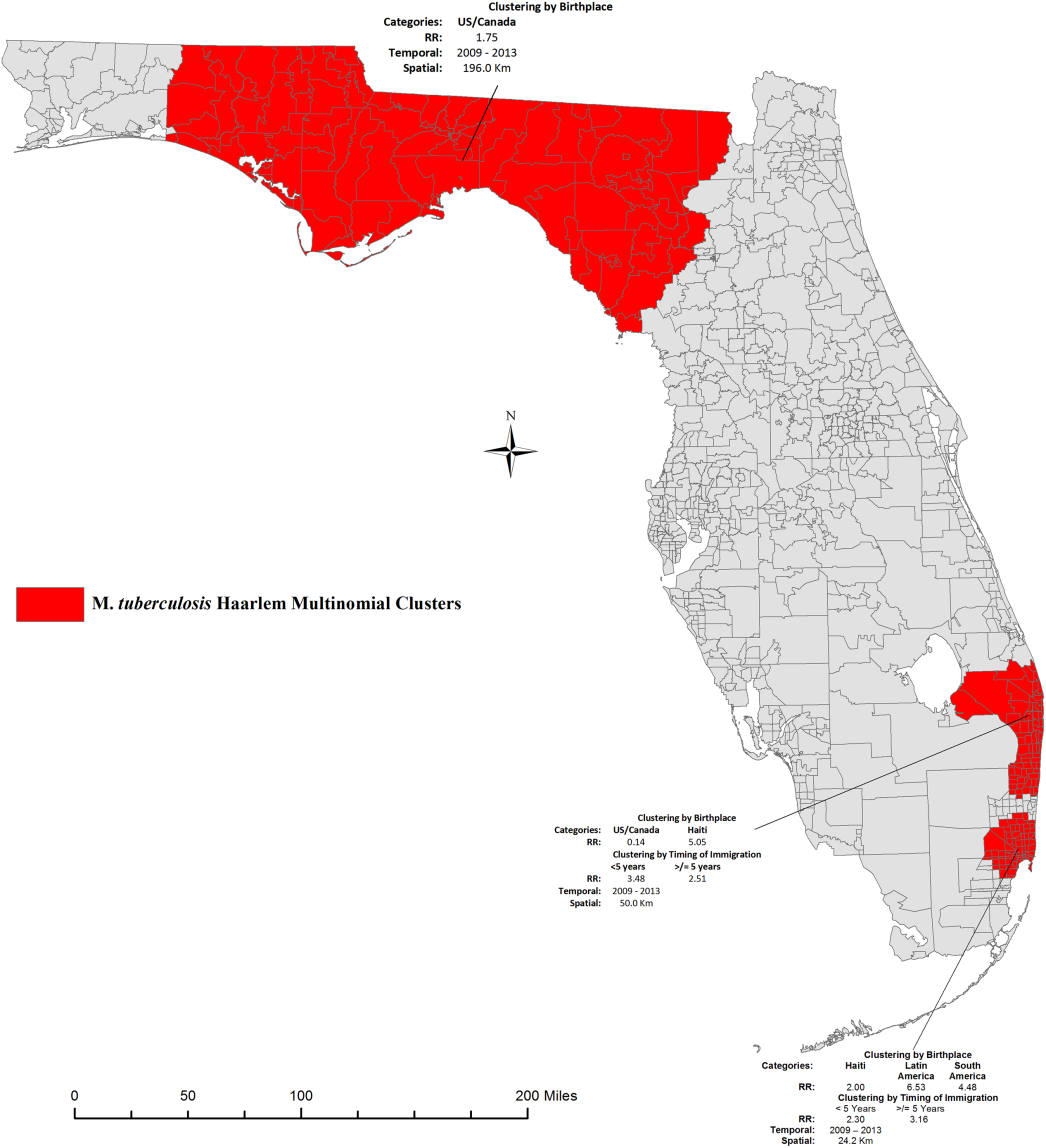
Multinomial space-time cluster of the M. *tuberculosis* Haarlem in Florida, 2009–2013. Maps show the location of significant multinomial space-time clusters of M. tuberculosis Haarlem in Florida, 2009–2013. Categories refer to case country/region of birth and timing of immigration among foreign-born cases. Relative Risk (RR) estimates indicate whether or not greater than expected numbers occurred in a given category. RR>1 represents a greater than expected number of individuals of certain category inside the spatial cluster compared to outside. Base map layer reprinted from [28] under a CC BY license, with permission from University of Florida GeoPlan Center, original copyright 2012.

The multinomial models showed three areas of high occurrence of the Haarlem sublineage; one which expanded over the Northern region of the state and the other two localized in the Southeast Region (Fig 3). The characteristics of the cases included inside each of these clusters are presented in Table 3. The Northern cluster included a total of 50 cases all born in the U.S./Canada (RR = 1.75). Fifty percent of the strains were of the H2 clade, 48.0% of the H1, and 2.0% were of the H3 clade. Four percent of the strains were resistant to at least one first-line anti-tuberculosis drugs (Isoniazid, Rifampin, and Ethambutol), a quarter of the cases were HIV positive and 56.0% were incarcerated at the time of TB diagnosis. Among persons with a history of incarceration, infection with the H2 sublineage was significantly higher (89.3%) compared to the other sublineages (p < .0001), while there was no significant difference in HIV and drug resistance prevalence between the three clades. The two Southern clusters (A and B) included a total of 66 cases. Over 90.0% of the 35 cases in Cluster A were born in Haiti (RR = 5.05); 60.0% had immigrated to the U.S. more than five years prior to diagnosis (RR = 2.51) and 29% had immigrated less than five years prior to diagnosis (RR = 3.48). Thirty percent of cases within cluster A were HIV positive and 5.7% were incarcerated at the time of diagnosis. Over 34% of the cases were infected with an H1 clade, 17.1% H2 and 48.6% H3. About 14.0% of the strains were resistant to at least one first-line TB drugs. Within Cluster B, there were greater than expected occurrence of foreign-born individuals from Latin America (RR = 6.53), South America (RR = 4.48) and Haiti (RR = 2.00) as compared to outside the cluster. In addition, the relative risk of cluster membership was 2.3 among recent immigrants and 3.16 among immigrants who had been in Florida at least five years prior to diagnosis. About 16% of the strains were of the H1 clade while 25.8% and 48.4% were of the H2 and H3 clades respectively. Resistance to at least one anti-tuberculosis drug was 9.7% and 19.4% of the cases were HIV positive. Prevalence of HIV, drug resistance or history of incarceration did not differ by infection with either one of the three Haarlem clades in either of the two Southern clusters. Based on the multinomial scan results, recent transmission was estimated at 46.40% (116/250) overall; 36.81% (60/163) among U.S-born cases, 62.07% (18/29) among recent immigrants and 65.52% (38/58) among immigrants who have lived in Florida at least five years.

**Table 3:** Characteristics of Cases inside the Multinomial Haarlem Clusters.

Characteristics	Northern Cluster (N = 50)	Southern Cluster A (N = 35)	Southern Cluster B (N = 31)	P-value^¥^
Haarlem Clades				
H1	24 (48.0)	12 (34.3)	5 (16.1)	< .0001
H2	25 (50.0)	6 (17.1)	8 (25.8)	
H3	1 (2.0)	17 (48.6)	15 (48.4)	
Other	-	-	3 (9.7)	
Any Drug Resistance				
No	48 (96.0)	30 (85.7)	28 (90.3)	0.2097
Yes	2 (4.0)	5 (14.3)	3 (9.7)	
Patient HIV Status				
Negative	36 (72.0)	24 (68.6)	25 (80.7)	0.3637
Positive	12 (24.0)	7 (20.0)	6 (19.4)	
Refused Testing	2 (4.0)	4 (11.4)	-	
History of Incarceration				
No	22 (44.0)	33 (94.3)	31 (100.0)	< .0001
Yes	28 (56.0)	2 (5.7)	-	
Birth Origin				
U.S.-born	50 (100.0)	4 (11.4)	6 (19.4)	< .0001
Immigrant (<5 years)		10 (28.6)	8 (25.8)	
Immigrant (≥ 5 years)		21 (60.0)	17 (54.8)	

Notes:

^¥^ indicates Fisher’s Exact Test; two-tailed test; analyses were conducted using SAS v9.4 (Cary, NC, USA).

## Discussion

The M. *tuberculosis* complex population structure observed in this study reflects Florida’s demography in its diversity. Indeed, we identified 74 different sublineages using spoligotyping and 24-Locus MIRU-VNTR in this study. Nevertheless many of the strain families were monomorphic at most of the loci tested while some were quite diverse, illustrating the contrasting evolutionary history of these loci within the foreign-born and U.S.-born populations in the State. Florida is a popular immigration destination for nations in Latin America, South America and the Caribbean, many with a national TB incidence several magnitude higher than that of Florida [36]. The spatial distribution of allelic diversity observed in this study is consistent with prior reports that have shown little contribution of Foreign-born population to TB transmission in the U.S. [37,38].

We estimated that close to five percent of TB cases reported in Florida during 2009–2013 was potentially due to recent transmission. In addition, among foreign-born persons, the relative risk of cluster membership was equally likely between recent (< 5 years) and long term (≥5 years) immigrants from Haiti, Latin America and South America. We identified potential transmission clusters of the Haarlem family spanning different regions of the state. In addition, these space-time clusters remained significant even after adjusting for both foreign-born population density and county-level HIV risk. These observations support conclusions that these clusters are not a result of common MTBC genotype reactivation due to HIV infection in Foreign-born population hubs in Florida and thus strengthen the evidence for M. *tuberculosis* Haarlem recent transmission in the state. Our findings are consistent with studies that have documented the circulation of the Haarlem sublineage in high TB incidence settings [39–41] as well as among immigrant groups in the US [3,8,42]. Clustered TB cases among recent immigrants are often interpreted as disease acquired prior to immigration [38], which does not negate the possibility of active TB transmission within recent immigrant groups in the U.S [43]. Of the 18 recent immigrants with genotype and space-time clustered disease in our study, five had lived in the US at least two years prior to diagnosis, which falls well within the estimated time for clinical presentation of recently acquired disease [44]. The dichotomy between recent and long-term immigrant highlights missed opportunities for effective TB control among foreign-born persons in the U.S.

Haarlem is a member of the “modern” MTBC lineages, and along with the ubiquitously successful Beijing strain, is associated with increased virulence and enhanced transmissibility as compared to the “ancient” MTBC lineages [45]. Interestingly, we identified space-time clusters of Haarlem genotypes clusters in regions of Florida where genetic diversity is also relatively high (S1 Fig). The results could signify that among the pool of circulating MTBC genotypes in the State, these Haarlem clades are more successful. As an obligate pathogen, MTBC success is intrinsically tied to its ability to establish initial infection and generate secondary cases [45]. As this study was cross-sectional and the clusters identified were small, we lacked the statistical power to investigate factors driving the selection of Haarlem clades in Florida. Future studies comparing the secondary infection rates of these genotypes and their capacity to cause lung cavitation as proxy measures for virulence are warranted.

Comparing the clinical characteristics of the multinomial Haarlem clusters identified in this study, we observed that resistance to first-line anti-tuberculosis drugs was significantly higher in the Southern clusters predominated by Foreign-born persons compared to the Northern cluster, while HIV prevalence was similar in both clusters. Our findings are not surprising as initial drug resistance to first-line anti-tuberculosis drugs is expected to be higher in individuals born in countries where TB treatment is at first empirical and infrastructure for drug susceptibility testing is lacking [46]. HIV and anti-tuberculosis drug resistance have been cited as two contemporary drivers of MTBC genetic diversity, as they may act together to increase patient infectious period when drugs to not work and increase transmissibility of strains to immunocompromised HIV infected persons [47]. However, there is inconclusive evidence as to whether HIV co-infected persons transmit more TB than HIV-negative individuals or whether drug resistance mutations confer a fitness advantage or disadvantage [47]. In our study we did not observed a significant difference in HIV and drug resistance prevalence between the different Haarlem sublineages, indicating that transmission was independent of HIV infection and not affected by drug resistance mutations. Prior studies that have documented the increased transmission of multidrug resistant Haarlem strains in South African children and HIV negative Tunisians [48,49]. Nevertheless, as our study was a cross-sectional analysis, we lack the evidence to conclusively comment on the possible transmission of multidrug resistant Haarlem strains in Florida.

The Haarlem sublineage is among the most geographically widespread modern MTBC sublineages and is found ubiquitously throughout North America and the Caribbean; nevertheless, the geographical distribution of certain clades may be more restricted [50]. In the US, the H2 and H3 clades are consistently reported in states where high proportion of the population is foreign-born [3,51]. The striking difference in H2 clade distribution and patient clinical characteristics observed between the multinomial Haarlem clusters in this study may be a result of host-pathogen co-evolution fostering more efficient MTBC transmission within population groups from the same region of birth [52,53]. MTBC transmission in urban centers where foreign and local populations interact tends to occur predominantly among high risk individuals or those with impaired immune systems [53]. A significantly higher proportion of individuals infected with an H2 strain in the Northern cluster had a history of incarceration and HIV prevalence within the three clusters was twenty percent and higher. Haarlem sublineage is among the predominant MTBC genotypes described in Haiti and among Haitian immigrants [54,55]. Prior molecular investigations in our laboratory had identified the H3 clade as emergent, i.e. spreading faster than background transmission rate, in the Haitian population living in Florida, with history of incarceration as the primary risk factor for transmission (unpublished data). It is impossible to postulate as to the transmission link between these three clusters using the current molecular markers. Nevertheless, it is likely that a combination of poor TB screening practices in Florida jails and HIV-induced immunosuppression played a role in the spread of the Haarlem clades in Florida.

The strength of these analyses is that we used a combination of two biomarkers to track MTBC genotype clusters in space and time over a period of 5 years in Florida. Combining spoligotyping and 24-locus MIRU-VNTR increased our discriminatory power to detect true MTBC genotype clusters, indicative of recent transmission. In addition, the five-year time period allowed for enough time to capture recent transmission that resulted in active disease reported to FDOH. It is nevertheless important to point out some important limitations of the analyses. First, we used surveillance data, which may suffer from reporting bias. Although by statute, TB is a reportable disease in Florida, it is possible that not all cases are captured. In particular, some cases diagnosed by private laboratories in 2009 were not genotyped and not included in these analyses. In addition, only culture confirmed cases are genotyped; thus, cases diagnosed based on clinical criteria alone and culture negative cases do not figure in these analyses. We observed a statistically significant difference in genotyped cases compared to reported cases among persons with a history of incarceration and country of birth. These findings may reflect a bias in genotyping due to outbreak investigations in the State of Florida. Indeed there have been a number of high profile TB outbreaks in the State that may have resulted in increased genotyping surveillance [56,57]. It is also likely that the difference reflects the decreasing trend in TB incidence in the general U.S. population but the higher burden among high risk groups, such as those with a history of incarceration, homelessness, or drug use [58–60]. We were concerned that the genotyping coverage for the State of Florida may not be representative of the reported culture-confirmed cases. This bias was formally evaluated using spatial descriptive statistics (S2 Fig). Although shift in the data could be observed for 2012, overall, there was little difference in dispersion (standard distance) or directionality (deviational ellipse), as evidenced by the high level of overlap in the circles and ellipses. In fact, we think the shift observed in 2012 could be attributed to decreasing TB incidence and ensuring change in TB epidemiology in Florida, whereby more and more of TB cases are reported within foreign-born individuals who tend to live in urban centers in Central and South Miami. In areas of low TB transmission and high level of foreign-born population, it is expected that MTBC genetic diversity will be high and representative of the population distribution. As genotyping coverage was low in some areas of the state, genetic diversity may be higher than estimated in this study. However, the spatial distribution of the reported and genotyped cases for each of the five years spanning the study period showed that genotyping efforts were equally distributed throughout the state and followed a similar spatial pattern as the case report data. Thus the MTBC genetic diversity reported in this study is representative of the reported TB cases in Florida during 2009 and 2013. An inherent limitation of the SaTScan methods is that it assumes a constant radius for each candidate spatial-temporal window, which effectively limits clusters in space and time. In reality, TB outbreaks tend to disperse in space as they progress, thus a conical rather than a cylindrical window would be more appropriate to simulate this natural TB outbreak progression. SaTScan, nevertheless, has been shown to be very sensitive to low disease incidence, as is the case in this study and tend to overestimate rather than underestimate cluster sizes, as compared to other spatial cluster detection methods [61]. Finally, as the multinomial space-time models were not adjusted for foreign-born population density or county-level HIV risk, we cannot refute the alternative explanation that the Haarlem clades are widespread throughout North America and the Caribbean and that cluster membership reflects the spatial distribution of the US and Foreign-born populations in Florida. Future studies involving more discriminatory molecular methods such as whole genome sequencing should help elucidate the link between these Haarlem clusters by identifying true transmission links and directionality [6,11].

Substantial progress has been made in controlling TB in the US; however, public health officials should not be complacent. Potential transmission clusters of Haarlem sublineages in areas of high genetic diversity raise concern over the clonal expansion of these globally successful strain families in Florida. In particular, renewed efforts to control TB in the prison population is warranted, as the prison system seems to be a common ground where Foreign and U.S.-born persons interact and high levels of HIV infection render persons susceptible to TB infection. In these settings, transmission bottlenecks can easily facilitate the predominance of these potentially more virulent Haarlem clades. Our findings can be used to inform TB control in Florida prisons and target interventions to Foreign-born populations in the community. In particular, renewed efforts to screen for TB infection in the jails prior to prison transfers are warranted to prevent outbreak in this vulnerable population.

## Supporting Information

S1 Fig Genotype Clusters in areas of Low and High Genetic Diversity in Florida, 2009–2013. Map shows the Space-time and multinomial genotype clusters of the Haarlem sublineage projected on to the allelic diversity map for Florida. Significant transmission clusters can be observed in areas of medium to high allelic diversity. Base map layer reprinted from [28] under a CC BY license, with permission from University of Florida GeoPlan Center, original copyright 2012.
(TIF)

S2 Fig The spatial means, 1-standard deviation ellipses and standard distances of the culture confirmed and genotyped cases for each of the five years (2009–2013). Descriptive statistics were calculated using zip code centroid for each database TIMS (Tuberculosis Information Management System) and GIMS (Genotype Information Management System) for each year. Overall, little difference in dispersion (standard distance) or directionality (deviational ellipse) was observed between the two datasets. Shifts in the spatial mean for the year 2012 can be observed, likely driven by decreasing incidence and changes in TB epidemiology in the State of Florida. Base map layer reprinted from [28] under a CC BY license, with permission from University of Florida GeoPlan Center, original copyright 2012.
(TIF)

S1 File Permission for copyrighted figure. Permission to reprint the base map layer of Florida under a CC BY license.
(PDF)

S1 Table Allelic Diversity of Different M. *tuberculosis* Complex Sublineages Isolates in Florida, 2009–2013. Table shows the allelic diversity for the each of the 24-MIRU-VNTR Loci examined in this study. While diversity at some locus was high (*h* >0.40), most sublineages where monomorphic (0.00 > *h* < 0.30).
(XLSX)
