# Supplementary figures and images for: Spatiotemporal Clustering of Mycobacterium tuberculosis Complex Genotypes in Florida: Genetic Diversity Segregated by Country of Birth
Source: PLoS One. 2016 Apr 19;11(4):e0153575. doi: 10.1371/journal.pone.0153575 (PMC4836742; doi:10.1371/journal.pone.0153575)

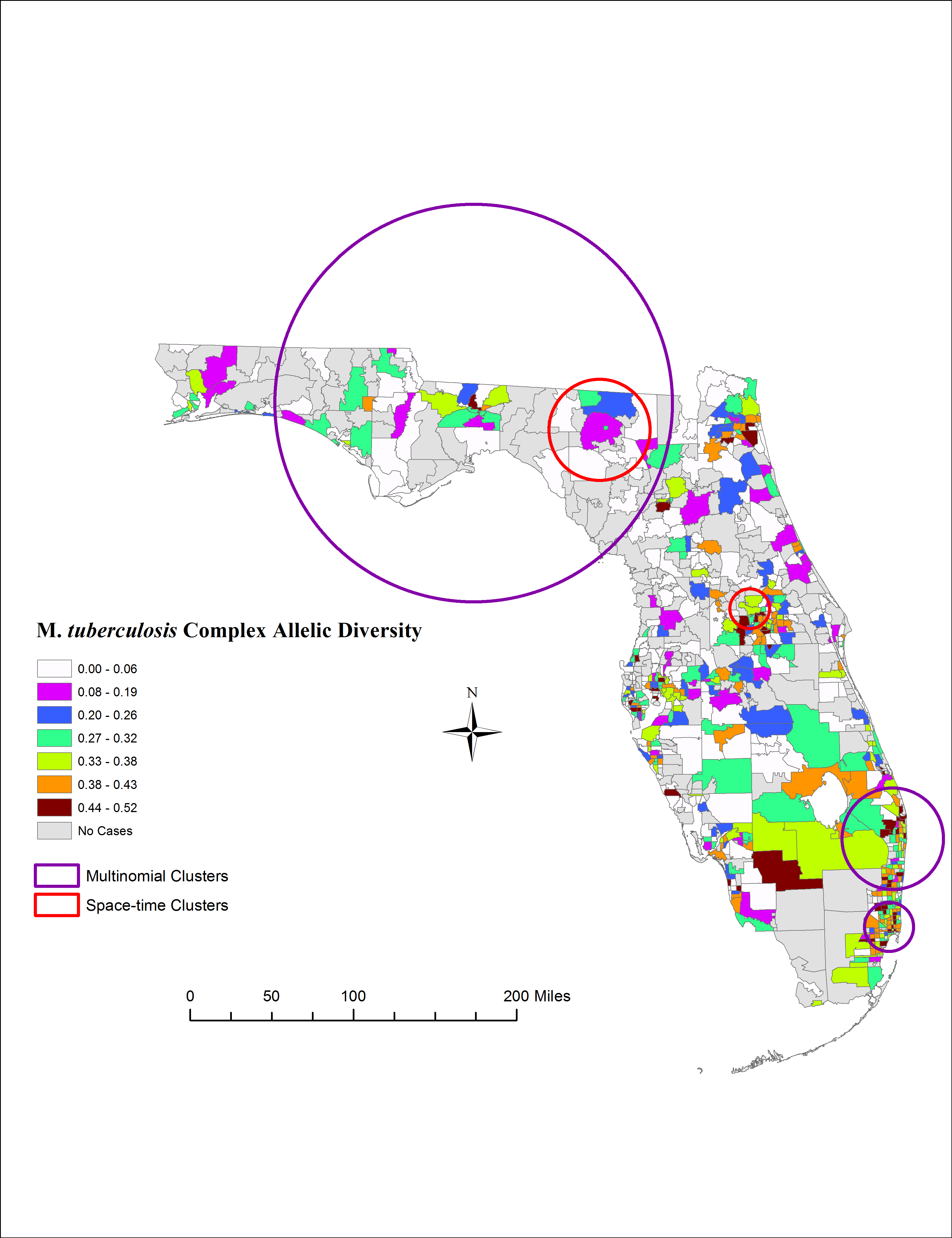

Supplement: S1 Fig — Map shows the Space-time and multinomial genotype clusters of the Haarlem sublineage projected on to the allelic diversity map for Florida. Significant transmission clusters can be observed in areas of medium to high allelic diversity. Base map layer reprinted from [28] under a CC BY license, with permission from University of Florida GeoPlan Center, original copyright 2012. (TIF) [file pone.0153575.s001.tif]

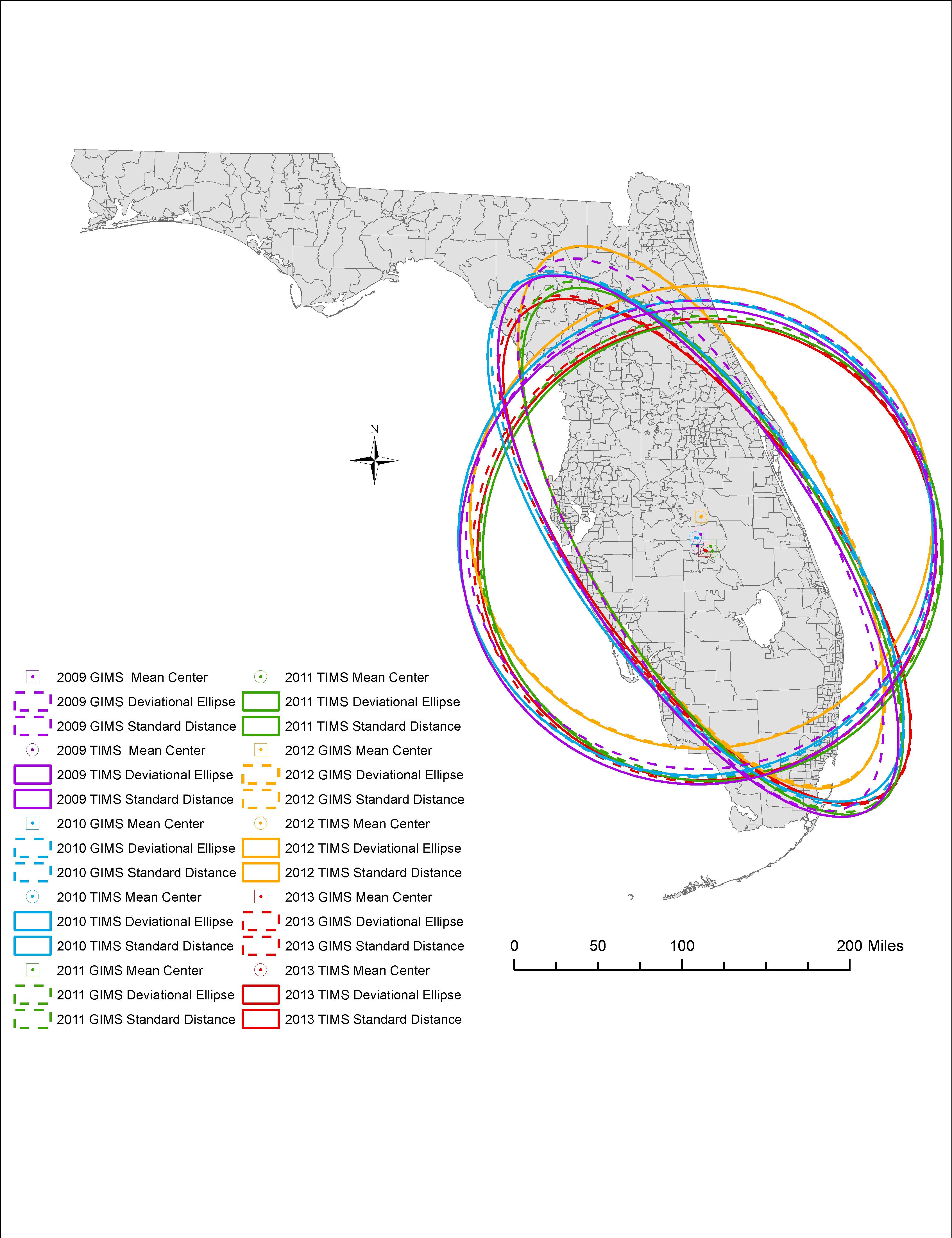

Supplement: S2 Fig — Descriptive statistics were calculated using zip code centroid for each database TIMS (Tuberculosis Information Management System) and GIMS (Genotype Information Management System) for each year. Overall, little difference in dispersion (standard distance) or directionality (deviational ellipse) was observed between the two datasets. Shifts in the spatial mean for the year 2012 can be observed, likely driven by decreasing incidence and changes in TB epidemiology in the State of Florida. Base map layer reprinted from [28] under a CC BY license, with permission from University of Florida GeoPlan Center, original copyright 2012. (TIF) [file pone.0153575.s002.tif]
