# Supplementary material for: Spatiotemporal Clustering of Mycobacterium tuberculosis Complex Genotypes in Florida: Genetic Diversity Segregated by Country of Birth
Source: PLoS One. 2016 Apr 19;11(4):e0153575. doi: 10.1371/journal.pone.0153575 (PMC4836742; doi:10.1371/journal.pone.0153575)
Supplement: S1 File — Permission to reprint the base map layer of Florida under a CC BY license. (PDF) [file pone.0153575.s003.pdf]

Re: Permission to Publish ZIPBND_2012 under CC BY 4.0 License

Sam Palmer <sam@geoplan.ufl.edu>

Thu 07-Jan-16 3:22 PM

To: Seraphin,Marie N <nseraphin@ufl.edu>;

You have our permission to use ZIPBND_2012 under the Creative Commons Attribution License

On 1/7/2016 3:10 PM, Seraphin,Marie N wrote:

> Hello,
>
> I request permission for the open-access journal PLOS ONE to publish ZIPBND_2012 under the Creative Commons Attribution License (CCAL) CC BY 4.0 (http://creativecommons.org/licenses/by/4.0/). Please be aware that this license allows unrestricted use and distribution, even commercially, by third parties. Please reply and provide explicit written permission to publish ZIPBND_2012 under a CC BY license.
>
> Thank you
>
> Marie
>
> Marie Nancy Seraphin, MPH
> PhD Student in Epidemiology
> College of Public Health and Health Professions
> College of Medicine
> University of Florida
> Email: nseraphin@ufl.edu
> Phone: 352-215-9472

--


```
***************************************************
Sam Palmer              GeoPlan Center
GIS Specialist          University of Florida
(352)392-3246           431 Architecture Bldg
sam@geoplan.ufl.edu     Gainesville, FL 32611
***************************************************
```
